# Population-Referenced Percentiles for Waist-Worn Accelerometer-Derived Total Activity Counts in U.S. Youth: 2003 – 2006 NHANES

**DOI:** 10.1371/journal.pone.0115915

**Published:** 2014-12-22

**Authors:** Dana L. Wolff-Hughes, David R. Bassett, Eugene C. Fitzhugh

**Affiliations:** Dept. of Kinesiology, Recreation & Sports Studies, The University of Tennessee, Knoxville, Tennessee, United States of America; University of Bremen, Germany

## Abstract

**Background:**

The total activity volume performed is an overall measure that takes into account the frequency, intensity, and duration of activities performed. The importance of considering total activity volume is shown by recent studies indicating that light physical activity (LPA) and intermittent moderate-to-vigorous physical activity (MVPA) have health benefits. Accelerometer-derived total activity counts (TAC) per day from a waist-worn accelerometer can serve as a proxy for an individual's total activity volume. The purpose of this study was to develop age- and gender-specific percentiles for daily TAC, minutes of MVPA, and minutes of LPA in U.S. youth ages 6 – 19 y.

**Methods:**

Data from the 2003 – 2006 NHANES waist-worn accelerometer component were used in this analysis. The sample was composed of youth aged 6 – 19 years with at least 4 d of ≥ 10 hours of accelerometer wear time (*N* = 3698). MVPA was defined using age specific cutpoints as the total number of minutes at ≥4 metabolic equivalents (METs) for youth 6 – 17 y or minutes with ≥2020 counts for youth 18 – 19 y. LPA was defined as the total number of minutes between 100 counts and the MVPA threshold. TAC/d, MVPA, and LPA were averaged across all valid days.

**Results:**

For males in the 50^th^ percentile, the median activity level was 441,431 TAC/d, with 53 min/d of MVPA and 368 min/d of LPA. The median level of activity for females was 234,322 TAC/d, with 32 min/d of MVPA and 355 min/d of LPA.

**Conclusion:**

Population referenced TAC/d percentiles for U.S. youth ages 6-19 y provide a novel means of characterizing the total activity volume performed by children and adolescents.

## Introduction

Physical activity (PA) in youth is associated with a reduced risk of obesity, type 2 diabetes, and other cardiometabolic risk factors [1]. The national PA guidelines recommend that children and youth accumulate 60 minutes/day (min/d) of moderate-to-vigorous PA (MVPA) to achieve substantial health benefits [2], [3]. Thus, in recent years researchers have attempted to use objective monitors to quantify the amount of time spent in MVPA.

Accelerometers have gained popularity with researchers, since they provide an objective means of assessing ambulatory PA. However, there is a valid concern that differences in accelerometer data collection and reduction methods (*i.e.,* placement site, number of axes, and cut-point selection) result in inconsistent estimates of PA. It is well known that when accelerometers are placed on different body sites, there is a discrepancy in the activity counts recorded [4]–[6]. In addition, it is known that tri-axial activity counts from a wrist accelerometer predict energy expenditure better than uni-axial activity counts [4], [5], [7]. For accelerometers placed at the waist, there is less evidence that tri-axial counts per minute are superior to uni-axial. Eston et al. [6] concluded that children's energy expenditure was more closely related to tri-axial counts (*r* = 0.91) than to uni-axial counts(*r* = 0.89) at the waist, but the difference was small. More recently, Sasaki et al. [8] developed cut-points for the ActiGraph GT3X worn at the waist, and concluded that further research was needed to determine whether vector magnitude (*i.e.*, tri-axial activity) counts yield better predictions of energy expenditure than vertical axis activity counts.

Another issue is the selection of cut-points that are applied to the accelerometer output in order to the classify PA intensity of youth. Specifically, Trost et al. [9] examined five youth-specific sets of cut-points [10]–[14], developed to classify the intensity of activity counts accumulated in the vertical axis of waist-worn ActiGraph accelerometers. The five methods underestimated the amount of time youth spent in MVPA by 39 – 74%. In addition, the various cut-points produce vastly different prevalence estimates of youth meeting the PA guidelines. For example, Loprinzi et al. [15] found the prevalence of children meeting the youth PA guidelines ranged from approximately 6 to 59%, depending on the cut-point selected. Furthermore, the various youth cut-points are problematic because there is no consensus regarding the appropriate selection of cut-points, and this lack of standardization prevents researchers from comparing results across studies.

Yet another issue is that most accelerometer-based studies in youth have focused on MVPA, but recent research suggests that light PA (LPA) also has positive effects on the cardiometabolic health of youth. For example, Carson et al. [16] found that LPA was associated with lower diastolic blood pressure and increased high-density lipoprotein cholesterol, independent of MVPA. Furthermore, recent research indicates MVPA accumulated in brief, intermittent bouts has similar associations with cardiometabolic health as MVPA accumulated in bouts of ≥10 min [17], [18]. Children especially tend to accumulate PA in brief, intermittent bouts, as opposed to long, continuous bouts of activity [19], [20].

Accelerometer-derived total activity counts per day (TAC/d) can provide a partial solution to these issues. TAC/d is a more direct expression of what the accelerometer measures and it is a proxy for the total activity volume, encompassing the frequency, intensity and duration of activity bouts. TAC/d is also a metric that could provide a standardized measure of accelerometer-derived PA in youth and allow for comparisons to be made between studies. In addition, although TAC/d has no intuitive meaning, it can be converted to percentiles that are both age- and gender-specific, which is important given the changes in energy expenditure that occur during growth and maturation. The percentiles approach has proven to be valuable for expressing anthropometric and fitness characteristics of youth, but it has not been applied to physical activity. Thus, the purpose of this study was to develop age- and gender-specific percentiles for TAC/d, MVPA, and LPA in U.S. youth ages 6 – 19 y.

## Methods

### Ethics Statement

The National Center for Health Statistics ethics review board approved the original survey protocols and consent was obtained from all participants and their parents/legal guardian if under 18 y. The University of Tennessee institutional review board approved the use of NHANES data in the present analysis.

### Participants

Data from the 2003 – 2006 National Health and Nutrition Examination Survey (NHANES) were used in this analysis. The NHANES uses a complex, multistage probability design to obtain a representative sample of the non-institutionalized U.S. population. The NHANES data are collected during an in-person home interview and a visit to a mobile examination center. During the interview, each participant's demographic, socioeconomic, and health-related information is collected. The examination consists of laboratory tests and medical and physiological measurements.

The present study was limited to youth, 6 – 19 y, with accelerometer data (*N* = 6770). Participants who did not have ≥ 4 d with ≥ 10 h of accelerometer wear time (*n* = 2135) were excluded from the analysis, resulting in the final sample (*N* = 3698).

### Physical Activity

Ambulatory participants six years of age and older were instructed to wear an ActiGraph model 7164 accelerometer on their right hip for seven days while awake, and to remove it when swimming or bathing [21]. The device was programmed to record vertical acceleration in 1-min epochs. The vertical accelerations captured by the device were filtered, full-wave rectified, and integrated over time, resulting in “activity counts” that correspond to the intensity of ambulatory activity [22]. Details of the accelerometer protocol can be found on the Centers for Disease Control and Prevention (CDC) website [23].

Accelerometer data were analyzed using the SAS macro provided by the National Cancer Institute website [24]. Non-wear time was defined as ≥60 consecutive minutes with zero accelerometer counts, allowing up to two minutes with limited movement [<100 counts/min (cpm)]. Daily wear time was determined by subtracting non-wear time from 24 h. To provide consistency with previous studies using NHANES accelerometer data, a valid day was defined as having ≥ 10 h of monitor wear [21], [25]. Only participants with at least four valid days of monitor wear time were included in this analysis. For participants between 6 – 17 y, the age-specific criteria described by Freedson et al. [26] were used with MVPA defined as the total number of minutes with counts above ≥ 4 metabolic equivalents (METs). For participants between 18 – 19 y, we used the threshold described by Troiano et al. [21], and MVPA was defined as the total number of minutes with ≥ 2020 cpm. LPA was defined as the total number of minutes between 100 counts and the MVPA threshold (*i.e.,* 4 METs or 2020 counts) and TAC/d represented the total counts accumulated daily. In the present analysis, MVPA, LPA, and TAC/d were averaged across all valid days.

### Statistical Analysis

Age-adjusted prevalence estimates and weighted means were calculated using SAS-callable SUDAAN 11.0 (Research Triangle Park, NC) and accounted for the complex sampling design using sample weights calculated according to NHANES analytical guidelines [27]. Smoothed, sex- and age-specific percentile curves corresponding to the 5^th^, 10^th^, 25^th^, 50^th^, 75^th^, 90^th^, 95^th^, and 97^th^ were generated using LMS ChartmakerPro (LMS Chartmaker version 2.54) software, which adjusts for NHANES sample weights. The LMS method uses a Box–Cox power transformation to normalize a particular measure (*i.e.,* TAC/d, MVPA, or LPA) across age [28]. Penalized likelihood is used to fit the L (skewness/Box-Cox power), M (median), and S (coefficient of variation) parameters as cubic splines [28]. Models that minimize the deviance and provide a good fit for the LMS parameters are selected to achieve smooth curves.

## Results

The mean age of the sample was 12.0 (*SE* = 0.1) years and 50.8% were male. For males, the median activity level was 441,431 TAC/d, with approximately 53 min/d of MVPA and 368 min/d of LPA. The median level of activity for females was 234,322 TAC/d, with approximately 32 min/d spent in MVPA and 355 min/d of LPA. Please see S1–S6 Tables for percentile values.

**Figure 1:**
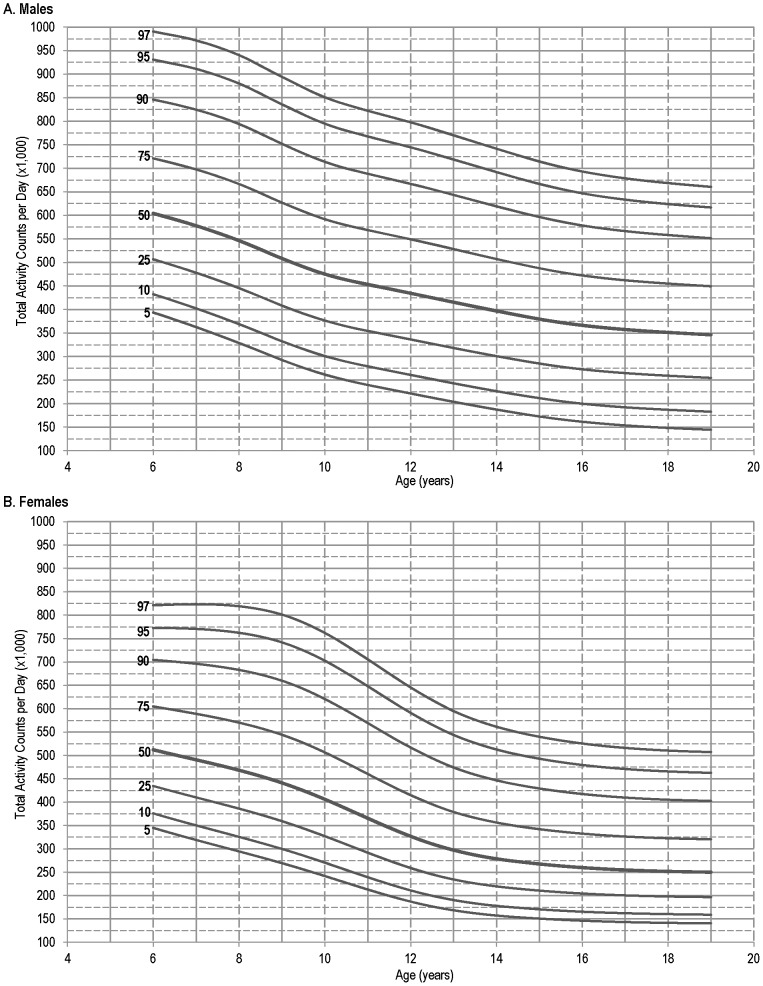
Percentiles of daily total activity counts per day for young males (A) and females (B), 6 – 19 years of age: 2003 – 2006 National Health and Nutrition Examination Survey. Note: Data were obtained with ActiGraph model 7164 worn at the waist.

Age- and gender-specific TAC/d percentiles are presented in Fig. 1. TAC/d was found to be higher in males than females, regardless of age. For both genders, an age-related decline in TAC/d was found across all percentiles. Specifically, the highest levels of TAC/d were seen at age six, with TAC/d declining with increasing age. An exception was seen for females in the 95^th^ and 97^th^ percentiles, where TAC/d was found to be highest at age seven.

**Figure 2:**
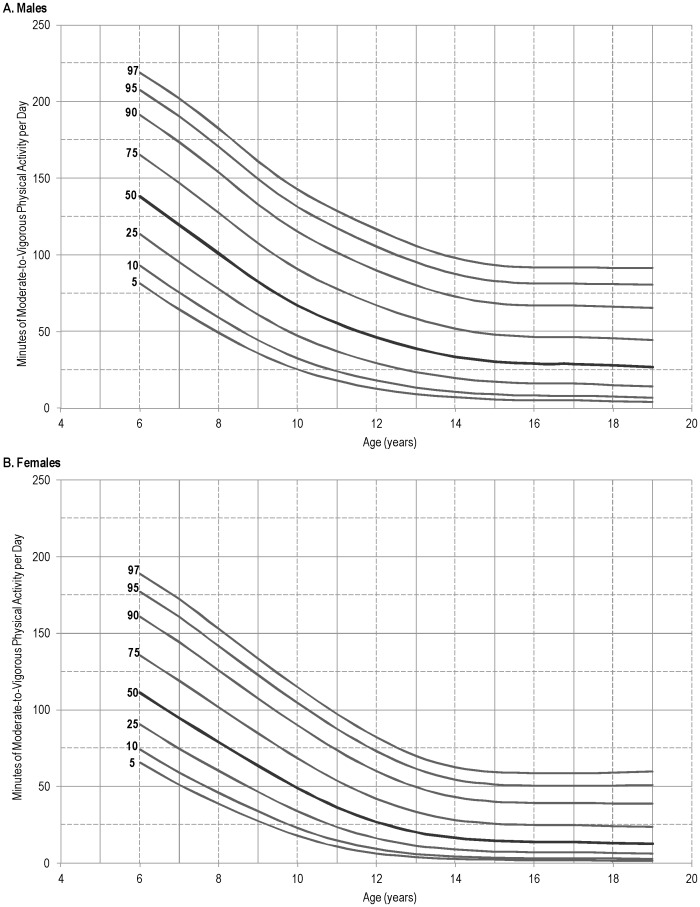
Percentiles of daily minutes of moderate-to-vigorous physical activity for young males (A) and females (B), 6 – 19 years of age: 2003 – 2006 National Health and Nutrition Examination Survey. Note: Data were obtained with ActiGraph model 7164 worn at the waist.

Fig. 2 displays the age- and gender-specific percentiles for MVPA. Across all percentiles and age groups, males accumulated greater amounts of MVPA then females. The gender difference also increased with age, with less than a 20% difference at age six and a 35 – 57% difference (depending on percentile) by 19 y. An age-related decline in MVPA was also seen in both genders. Specifically, MVPA decreased as age increased from 6 – 14 y, beyond which there was little or no change in MVPA (for both genders).

**Figure 3:**
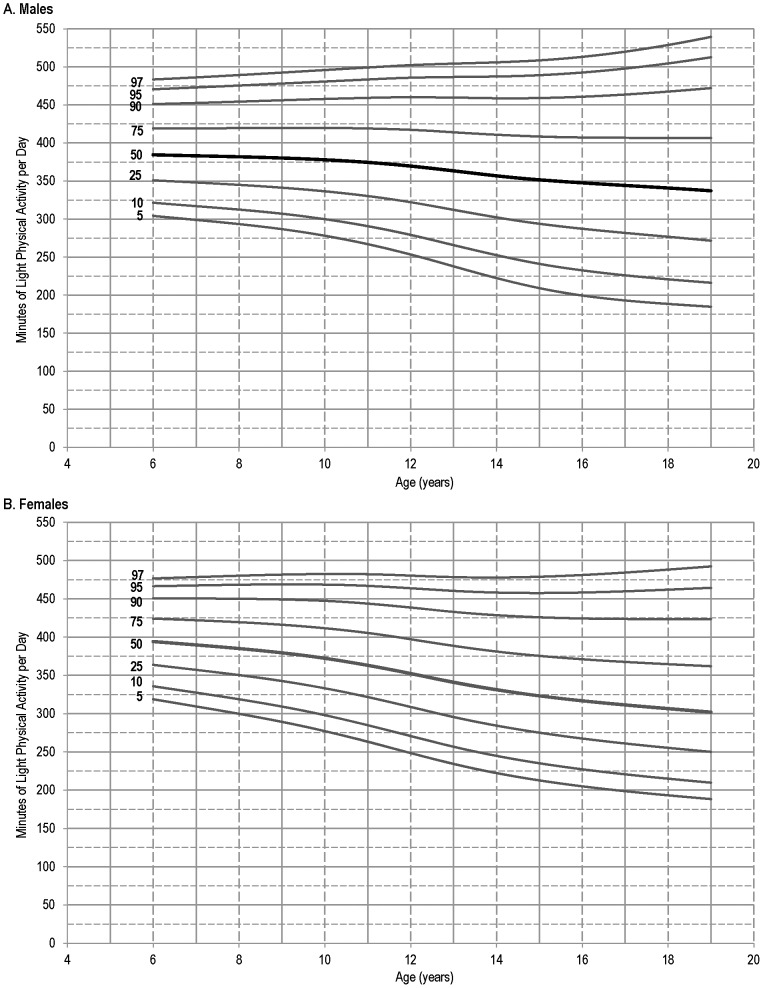
Percentiles of daily minutes of light physical activity for males (A) and females (B), 6 – 19 years of age: 2003 – 2006 National Health and Nutrition Examination Survey. Note: Data were obtained with ActiGraph model 7164 worn at the waist.

Fig. 3 presents age- and gender- specific percentiles for LPA. Females in the 5^th^ percentile had more LPA than males. Males in the 10^th^ to 75^th^ percentile accumulated more LPA than females between ages 9 – 19 y, and above the 90^th^ percentile males had greater volumes than females regardless of age. Below the 95^th^ percentile, an age-related decline was seen for both genders. For females in the 95^th^ and 97^th^ percentiles, however, LPA increased until age 11 above which it declined. In males above the 90^th^ percentile, an age-related increase in LPA was seen.

**Figure 4:**
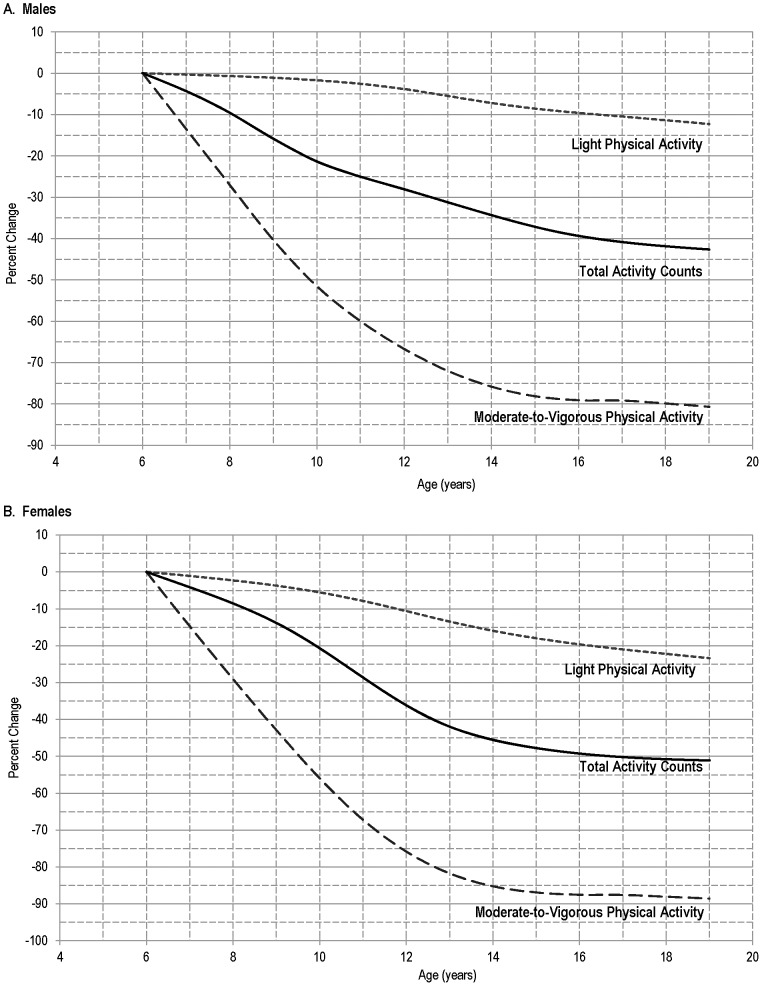
Relationship of accelerometer-derived physical activity levels and age, for males (A) and females (B), 6 – 19 years of age in the 50^th^ percentile: 2003 – 2006 National Health and Nutrition Examination Survey. Note: Data are expressed as percent change from the values seen in 6-year old children.

Fig. 4 compares the age-related change in TAC/d, MVPA, and LPA for the 50^th^ percentile of U.S. youth. Between 6 – 19 y, TAC/d decreased by approximately 43% in males and 51% in females. Minutes spent in LPA declined by 12% and 23% between 6 – 19 y in males and females, respectively. Minutes of MVPA showed the greatest age-related decline, with an 81% decline seen between 6 – 19 y in males, and an 89% decline seen in females between these ages.

## Discussion

This study is the first to report population-referenced data for TAC/d in a nationally representative sample of U.S. children and adolescents between the ages of 6 and 19 y. TAC/d provides researchers with a measure of the total activity volume, encompassing all PA intensity levels. Given recent studies indicating the benefits of LPA and short, intermittent bouts of MVPA in youth [16]–[18], researchers are beginning to recognize the merits of a PA metric that accounts for the total activity volume performed by individuals [29]–[32].

The total activity counts in this study are derived from the waist-worn ActiGraph model 7164 used in the NHANES 2003–2006, and are comparable to the vertical axis of the current generation ActiGraph (GT3X+) [8], [33], [34] that is widely used by many researchers today. One of the advantages of expressing the PA of youth in this way is that it provides an indication of how active an individual is, relative to other U.S. youth of the same age and gender. Furthermore, these results provide a useful means of showing how the total activity volume changes throughout childhood and adolescence.

Consistent with previous research, the results of this study indicate that boys are more active than girls and that there is a decline in PA throughout childhood and adolescence [21], [35]. In both genders, MVPA was found to decline greatly from ages 6 – 19 y while the decline in LPA was less severe. As TAC/d incorporates both LPA and MVPA, the rate of age-related decline in the median values of this metric fell in between the rates of decline in the other two PA metrics.

As previously noted, the *2008 Physical Activity Guidelines for Americans* [3] recommended that U.S. youth accumulate at least 60 minutes of MVPA. No minimum bout duration was specified for youth as it was for adults. The PA guidelines have spurred interest in measuring how many minutes of MVPA children perform, in order to determine whether or not they are meeting the PA guidelines. However, there are several factors complicating the use of accelerometers to assess time spent in various PA intensities [36]–[38]. The first factor is the proliferation of MVPA cut-points for youth. The second factor is the use of varying measurement periods; it has been shown that the decision to use 15-s, 30-s, or 60-s epochs has a large impact on the number of minutes spent in MVPA [39]–[41]. The lack of consensus on which cut-points and measurement period should be used, has led to a lack of standardization among research studies involving youth. In addition, there are other factors that contribute to lack of standardization, such as the PA measurement device used, the body site on which it is worn, and tri-axial vs. uni-axial cut-points.

TAC/d is an additional PA metric that has several strengths. Specifically, TAC/d is the most direct expression of what the accelerometer records, capturing both the intensity and pattern of ambulatory PA. TAC/d is a proxy for the total activity volume, with each minute weighted in proportion to the intensity of the activity. The inclusion of all intensities is important since previous research indicates both LPA [29] and non-bout MVPA [30] are associated with health benefits. Additionally, TAC/d could serve as a standardized measure of PA, allowing comparisons to be drawn across studies that employ a waist-worn ActiGraph.

The use of TAC/d also has a number of limitations. Specifically, the generalizability of these percentiles is limited to U.S. youth ages 6 – 19 y who have waist-worn ActiGraph accelerometer data. Also, the activity counts recorded by the uniaxial, waist-worn accelerometer used in this study do not adequately capture non-ambulatory activities (*e.g.,* cycling, weight training, and swimming). Another limitation is that activity counts are dependent on the PA monitor used. However, the ActiGraph 7164 is by far the most commonly used accelerometer in PA research [32], and it has been shown to provide reliable and valid assessments of PA [8], [42]. Furthermore, the ActiGraph 7164 yields similar data to the vertical axis of recent ActiGraphs with tri-axial accelerometers [8], [43]. One final limitation of TAC/d is that the NHANES changed from the waist location (in 2003–2006) to the wrist (in 2008–2011), raising a concern that the data reported in this paper could soon become obsolete if researchers follow the latest methods used by the federal government. However, the Risk Factor Monitoring and Methods branch of the National Cancer Institute (NCI) plans to use the TAC/d percentiles approach with the vector magnitude data from the wrist-worn ActiGraph GT3X data, indicating the value of this approach.

In conclusion, this paper describes population referenced TAC/d percentiles for U.S. children, ages 6 – 19 y. These percentiles provide PA, public health, and medical researchers with a method of characterizing the activity levels of children and adolescents using waist-worn accelerometers. Additionally, TAC/d can be reported alongside other measures of PA; thus, it complements, rather than replaces, other PA variables. If widely adopted, the use of TAC/d could help to standardize accelerometer-derived measurements of activity obtained from the waist and provide a novel means of comparing results between studies.

## Supporting Information

S1 Table **Percentiles for Total Activity Counts in US Boys Ages 6-19 (** ***N*** **= 1844).**
(DOCX)

S2 Table **Percentiles for Total Activity Counts in US Girls Ages 6-19 (** ***N*** **= 1815).**
(DOCX)

S3 Table **Percentiles for Minutes of Moderate-to-Vigorous Physical in US Boys Ages 6-19 (** ***N*** **= 1844).**
(DOCX)

S4 Table **Percentiles for Minutes of Moderate-to-Vigorous Physical in US Girls Ages 6–19 (** ***N*** **= 1815).**
(DOCX)

S5 Table **Percentiles for Minutes of Light Physical Activity in US Boys Ages 6–19 (** ***N*** **= 1844).**
(DOCX)

S6 Table **Percentiles for Minutes of Light Physical Activity in US Girls Ages 6–19 (** ***N*** **= 1815).**
(DOCX)
