# Supplementary material for: Population-Referenced Percentiles for Waist-Worn Accelerometer-Derived Total Activity Counts in U.S. Youth: 2003 – 2006 NHANES
Source: PLoS One. 2014 Dec 22;9(12):e115915. doi: 10.1371/journal.pone.0115915 (PMC4274159; doi:10.1371/journal.pone.0115915)
Supplement: S1 Table — Percentiles for Total Activity Counts in US Boys Ages 6-19 ( N = 1844). (DOCX) [file pone.0115915.s001.docx]

Table S1: Percentiles for Total Activity Counts in US Boys Ages 6-19 (*N*=1844).

Percentiles

Age L M S 5 10 25 50 75 90 95 97

6 -0.02 604189 0.26 393593 432544 506607 604189 720984 845710 930658 990435

7 0.05 577988 0.28 362714 402425 478120 577988 697415 824573 910871 971427

8 0.13 546317 0.30 329064 369084 445500 546317 666552 793941 879956 940086

9 0.20 508469 0.32 292892 332604 408497 508469 627149 752038 835819 894118

10 0.27 475165 0.34 261804 301161 376373 475165 591759 713504 794604 850769

11 0.32 452991 0.35 239845 279247 354494 452991 568536 688288 767548 822202

12 0.37 434693 0.36 221736 261211 336509 434693 549167 666957 744450 797673

13 0.42 415593 0.37 204049 243395 318324 415593 528251 643314 718553 770026

14 0.46 396862 0.39 187522 226610 300898 396862 507249 619162 691909 741489

15 0.50 379747 0.40 172896 211681 285224 379747 487748 596473 666759 714498

16 0.53 366367 0.40 161528 200086 273035 366367 472388 578486 646762 693003

17 0.55 357422 0.41 153865 192298 264881 357422 462091 566386 633282 678498

18 0.57 351369 0.41 148670 187028 259371 351369 455103 558151 624095 668606

19 0.58 346472 0.42 144485 182783 254926 346472 449428 551446 616608 660539
