# Supplementary material for: Population-Referenced Percentiles for Waist-Worn Accelerometer-Derived Total Activity Counts in U.S. Youth: 2003 – 2006 NHANES
Source: PLoS One. 2014 Dec 22;9(12):e115915. doi: 10.1371/journal.pone.0115915 (PMC4274159; doi:10.1371/journal.pone.0115915)
Supplement: S2 Table — Percentiles for Total Activity Counts in US Girls Ages 6-19 ( N = 1815). (DOCX) [file pone.0115915.s002.docx]

Table S2: Percentiles for Total Activity Counts in US Girls Ages 6-19 (*N*=1815).

Percentiles

Age L M S 5 10 25 50 75 90 95 97

6 -0.11 511960 0.25 344990 375889 434583 511960 604858 704597 772909 821183

7 -0.11 490650 0.27 318921 350171 410224 490650 588872 696111 770519 823535

8 -0.11 468091 0.29 294440 325555 385989 468091 569938 682856 762143 819065

9 -0.11 441214 0.31 269540 299879 359363 441214 544177 659915 742055 801428

10 -0.11 405993 0.32 241754 270428 327114 405993 506443 620732 702612 762156

11 -0.11 365455 0.34 212948 239300 291760 365455 460284 569298 648028 705576

12 -0.11 326813 0.35 187129 211064 258982 326813 414836 516875 591048 645493

13 -0.11 297126 0.36 168372 190324 234420 297126 378906 474180 543706 594866

14 -0.11 278859 0.36 157374 178046 219625 278859 356265 446621 512659 561300

15 -0.11 267533 0.36 150674 170539 210522 267533 342107 429242 492976 539943

16 -0.11 259797 0.36 146119 165431 204317 259797 332416 417321 479455 525259

17 -0.11 254908 0.36 143248 162209 200399 254908 326284 409770 470885 515947

18 -0.11 251915 0.36 141492 160238 198003 251915 322527 405142 465630 510235

19 -0.11 250205 0.36 140491 159114 196635 250205 320381 402497 462626 506970
