# Supplementary material for: Population-Referenced Percentiles for Waist-Worn Accelerometer-Derived Total Activity Counts in U.S. Youth: 2003 – 2006 NHANES
Source: PLoS One. 2014 Dec 22;9(12):e115915. doi: 10.1371/journal.pone.0115915 (PMC4274159; doi:10.1371/journal.pone.0115915)
Supplement: S3 Table — Percentiles for Minutes of Moderate-to-Vigorous Physical in US Boys Ages 6-19 ( N = 1844). (DOCX) [file pone.0115915.s003.docx]

Table S3: Percentiles for Minutes of Moderate-to-Vigorous Physical in US Boys Ages 6-19 (*N*=1844).

Percentiles

Age L M S 5 10 25 50 75 90 95 97

6 0.56 138 0.28 82 93 113 138 165 191 208 219

7 0.53 120 0.32 64 75 95 120 147 173 190 202

8 0.50 101 0.37 49 59 78 101 127 154 171 182

9 0.48 82 0.42 36 44 61 82 107 133 149 161

10 0.45 67 0.48 25 33 47 67 91 115 131 143

11 0.43 55 0.54 18 24 37 55 78 102 118 129

12 0.41 46 0.60 13 18 30 46 67 90 106 117

13 0.40 39 0.66 9 14 24 39 58 80 95 106

14 0.39 33 0.71 7 11 20 33 52 73 87 98

15 0.38 30 0.75 6 9 17 30 48 68 83 93

16 0.37 29 0.77 5 8 16 29 46 67 81 92

17 0.37 29 0.77 5 8 16 29 46 67 81 92

18 0.37 28 0.79 5 8 15 28 45 66 81 91

19 0.36 27 0.82 4 7 14 27 44 65 81 92
