# Supplementary material for: Population-Referenced Percentiles for Waist-Worn Accelerometer-Derived Total Activity Counts in U.S. Youth: 2003 – 2006 NHANES
Source: PLoS One. 2014 Dec 22;9(12):e115915. doi: 10.1371/journal.pone.0115915 (PMC4274159; doi:10.1371/journal.pone.0115915)
Supplement: S4 Table — Percentiles for Minutes of Moderate-to-Vigorous Physical in US Girls Ages 6–19 ( N = 1815). (DOCX) [file pone.0115915.s004.docx]

Table S4: Percentiles for Minutes of Moderate-to-Vigorous Physical in US Girls Ages 6-19 (*N*=1815).

Percentiles

Age L M S 5 10 25 50 75 90 95 97

6 0.26 111 0.30 66 74 91 111 136 161 177 189

7 0.29 95 0.34 51 59 75 95 119 144 161 172

8 0.31 79 0.39 39 46 60 79 102 126 142 153

9 0.33 64 0.45 28 34 46 64 85 107 123 133

10 0.33 49 0.52 18 23 34 49 68 90 104 115

11 0.32 37 0.61 11 15 23 37 54 74 88 97

12 0.31 27 0.71 6 9 16 27 42 60 73 82

13 0.29 20 0.79 4 6 11 20 33 50 61 70

14 0.27 16 0.86 3 4 9 16 28 43 54 63

15 0.26 15 0.91 2 4 7 15 26 40 51 60

16 0.26 14 0.93 2 3 7 14 25 39 51 59

17 0.25 14 0.93 2 3 7 14 25 39 50 59

18 0.25 13 0.96 2 3 7 13 24 39 50 59

19 0.23 13 0.99 2 3 6 13 24 39 51 60
