# Supplementary material for: Population-Referenced Percentiles for Waist-Worn Accelerometer-Derived Total Activity Counts in U.S. Youth: 2003 – 2006 NHANES
Source: PLoS One. 2014 Dec 22;9(12):e115915. doi: 10.1371/journal.pone.0115915 (PMC4274159; doi:10.1371/journal.pone.0115915)
Supplement: S5 Table — Percentiles for Minutes of Light Physical Activity in US Boys Ages 6–19 ( N = 1844). (DOCX) [file pone.0115915.s005.docx]

Table S5: Percentiles for Minutes of Light Physical Activity in US Boys Ages 6-19 (*N*=1844).

Percentiles

Age L M S 5 10 25 50 75 90 95 97

6 0.68 385 0.13 304 321 351 385 419 451 471 483

7 0.72 383 0.14 299 317 348 383 419 453 473 486

8 0.77 382 0.15 293 312 345 382 420 454 475 489

9 0.82 380 0.15 287 307 341 380 420 456 478 492

10 0.88 378 0.16 278 300 337 378 420 458 481 496

11 0.95 375 0.18 267 291 330 375 419 459 484 499

12 1.01 370 0.19 254 279 322 370 417 460 486 502

13 1.05 363 0.21 238 266 312 363 414 460 487 504

14 1.07 357 0.23 223 253 302 357 411 459 487 506

15 1.08 352 0.24 209 241 294 352 408 459 489 509

16 1.05 348 0.26 199 233 287 348 407 461 493 513

17 0.96 344 0.27 193 226 282 344 407 464 498 520

18 0.85 341 0.28 188 221 277 341 407 468 505 529

19 0.72 337 0.30 185 216 272 337 407 472 513 539
