# Supplementary material for: Population-Referenced Percentiles for Waist-Worn Accelerometer-Derived Total Activity Counts in U.S. Youth: 2003 – 2006 NHANES
Source: PLoS One. 2014 Dec 22;9(12):e115915. doi: 10.1371/journal.pone.0115915 (PMC4274159; doi:10.1371/journal.pone.0115915)
Supplement: S6 Table — Percentiles for Minutes of Light Physical Activity in US Girls Ages 6–19 ( N = 1815). (DOCX) [file pone.0115915.s006.docx]

Table S6: Percentiles for Minutes of Light Physical Activity in US Girls Ages 6-19 (*N*=1815).

Percentiles

Age L M S 5 10 25 50 75 90 95 97

6 1.23 394 0.11 319 336 364 394 424 451 466 477

7 1.17 390 0.12 309 327 357 390 422 450 467 478

8 1.11 385 0.13 300 319 350 385 419 450 468 480

9 1.04 379 0.14 289 309 343 379 416 449 469 482

10 0.96 372 0.16 277 298 333 372 412 447 469 482

11 0.86 363 0.17 263 285 322 363 405 444 467 482

12 0.77 352 0.19 249 271 309 352 397 439 464 480

13 0.67 341 0.20 234 257 296 341 389 433 460 478

14 0.57 331 0.22 222 245 284 331 381 429 458 478

15 0.48 323 0.23 213 235 275 323 375 426 458 479

16 0.41 317 0.24 205 227 267 317 371 424 458 481

17 0.34 311 0.25 199 221 261 311 368 424 460 484

18 0.27 306 0.26 193 215 255 306 365 423 462 488

19 0.21 302 0.27 188 210 250 302 362 424 464 492
